# Neoadjuvant immune checkpoint inhibitor in combination with chemotherapy or chemoradiotherapy in resectable esophageal cancer: A systematic review and meta-analysis

**DOI:** 10.3389/fimmu.2022.998620

**Published:** 2022-09-13

**Authors:** He Wang, Sihan Li, Tingting Liu, Jun Chen, Jun Dang

**Affiliations:** ^1^ Department of Radiation Oncology, The First Affiliated Hospital of China Medical University, Shenyang, China; ^2^ Department of Radiation Oncology, Anshan Cancer Hospital, Anshan, China; ^3^ Department of Radiation Oncology, Shenyang Tenth People’s Hospital, Shenyang, China

**Keywords:** esophageal cancer, neoadjuvant, immune checkpoint inhibitor, chemotherapy, chemoradiotherapy, pathological complete response, meta-analysis

## Abstract

**Background:**

Neoadjuvant immune checkpoint inhibitor in combination with chemotherapy (nICT) or chemoradiotherapy (nICRT) has been tested in resectable esophageal cancer. Nevertheless, efficacy and safety for this new strategy have not been clearly demonstrated.

**Patients and methods:**

PubMed, Embase, Cochrane Library, Web of Science, and scientific meetings were searched for eligible trials until June 30, 2022. The primary outcome of interest was pathological complete response (pCR). The random-effect model was used for statistical analysis.

**Results:**

Twenty-seven trials with 809 patients were identified. The estimated rates of pCR for nICRT and nICT were comparable (32.7%, 95% CI: 20.3%-45.1% vs 26.3%, 95% CI: 19.8%-32.8%; P = 0.37). As for safety, surgical resection rate, R0 resection rate, surgical delay rate, and surgical mortality rate were similar between nICRT and nICT, while more grade ≥3 treatment-related adverse events were observed for nICRT (52.6%, 95% CI: 30.7%-74.5% vs 19.9%, 95% CI: 8.8%-31.0%; P = 0.01). In subgroup analysis, nICRT achieved higher pCR rate compared to nICT (56.2%, 95% CI: 41.0%-71.3% vs 27.2%, 95% CI: 20.2%-34.1%; P < 0.001) for squamous cell carcinoma (SCC) but adenocarcinoma. In patients receiving nICT, PD-L1 expression CPS ≥1 showed higher pCR rate compared to CPS <1 (51.3%, 95% CI: 41.4%-61.2% vs 26.6%, 95% CI: 8.6%-44.5%; P = 0.02); regimen of paclitaxel plus carboplatin/cisplatin (PC/TP) and 3-4 cycles of nICT did not lead to an significantly improved pCR rate compared to other chemotherapy regimens and 2 cycles of nICT, respectively, despite without increased toxicity.

**Conclusion:**

Both nICT and nICRT achieved promising pCR rates with acceptable tolerability, and nICRT was likely to have more antitumor efficacy compared to nICT for patients with SCC. PD-L1 status seemed to be predictive of pCR in patients receiving nICT; pCR rate did not appear to be greatly affected by CT regimen and increasing cycles of nICT.

## Introduction

Esophageal cancer (EC) is the 7th most frequently diagnosed cancer and the 6th leading cause of cancer-related deaths in the world (1, 2). Esophageal squamous cell carcinoma (ESCC) and esophageal adenocarcinoma (EAC) are two main histological subtypes of EC. Currently, neoadjuvant chemoradiotherapy (nCRT) followed by surgery is a standard regimen for resectable EC based on the results of CROSS (3, 4) and NEOCRTEC501032 (5, 6) trials. However, the 5-year overall recurrence is still high (6), and long-term survival is unsatisfactory (4). Neoadjuvant chemotherapy (nCT) has been recognized as another standard treatment for resectable EC (7, 8). To date, there is still no clear evidence supporting a difference in survival benefit between nCRT and nCT (9, 10).

Given the superior efficacy and manageable toxicity of immune checkpoint inhibitor (ICI) in metastatic EC (11–13), there is an increasing interest in examining the addition of ICI to nCRT (nICRT) or nCT (nICT) in resectable disease. Initial findings from a number of phase 1 or 2 trials have supported the tolerability and/or antitumor efficacy of nICRT and nICT (14–40). Nevertheless, the superiority of this combination strategy remains uncertain due to lack of randomized control trials (RCTs) with long-term outcomes. Moreover, there are still outstanding questions such as the selection of nICRT or nICT and the ideal predictive biomarkers.

In light of these issues, we performed the systematic review and meta-analysis to assess antitumor efficacy and safety of nICRT and nICT. We used pathological complete response (pCR) as the primary outcomes of interest because they might be predictive of overall survival (OS) for patients with EC (41, 42).

## Methods

This systematic review and meta-analysis was conducted according to the Preferred Reporting Items for Systematic Reviews and Meta-Analyses (PRISMA) guidelines (43) (**Supplementary File: Table S1**), and was registered in INPLASY international platform of registered systematic review and meta-analysis protocols with registration number INPLASY202260052.

### Literature search

We searched PubMed, Embase, Cochrane Library and Web of Science for relevant publications until June 30, 2022, using the search terms (“esophageal cancer” or “oesophageal cancer” or “esophageal neoplasms” or “esophageal carcinoma”), and (“neoadjuvant” or “preoperative”), and (“immunotherapy” or “immune checkpoint inhibitors” or “PD-1/PD-L1 blockades” or “anti-PD-1/PDL1”). The search strategy in details is presented in **Supplementary File: Table S2**. Abstracts of recent important meetings were also inspected, including the American Society of Clinical Oncology (ASCO), European Society for Medical Oncology (ESMO), and American Society for Radiation Oncology (ASTRO). References of relevant studies were reviewed for additional articles.

### Inclusion and exclusion criteria

The inclusion criteria were as follows: (1) single-arm or multi-arm trials examining nICRT or nICT in resectable EC; (2) reported at least one of the following outcomes: pCR (defined as no viable tumor cells in the resected specimen, ypT0N0), surgical resection rate (the ratio of patients who underwent surgical resection to those who were planned to), R0 resection rate (the ratio of patients achieving a R0 resection to all patients undergoing surgical resection), surgical delay rate, incidence of surgical mortality rate, and incidence of grade ≥3 treatment-related adverse events (TRAEs) during neoadjuvant therapy. If multiple articles were published from the same trial, the most recent one which reported the most comprehensive data was selected.

### Data extraction

The following information was extracted independently by two authors (HW and SL): first author, publication year, design, region, sample size, histological type, intervention, and data regarding outcome measures.

### Quality assessment

Risk of bias of RCTs was independently assessed by two authors (HW and SL), using the Cochrane Risk of Bias Tool (44). The trials were finally classified as low (all domains indicated as low risk), high (one or more domains indicated as high risk), and unclear risk of bias (more than three domains indicated as unclear risk).

### Statistical analysis

The primary outcome of interest was pCR. The second outcomes of interest were surgical safety including surgical resection rate, R0 resection rate, surgical delay rate, and surgical mortality rate, and incidence of grade ≥3 TRAEs. The random effect model was used for statistical analysis, using the software R (version 3.5.3, R Foundation for Statistical Computing) *via* the meta package. The inverse variance method was used to calculate pooled estimates of the outcomes and their 95% confidence intervals (CIs). The Chi-square (χ^2^) and I-square (I^2^) test were performed to detect the presence of heterogeneity, and significant heterogeneity was considered present if P value of less than 0.10 or I^2^ greater than 50%. Subgroup analyses were performed according to histological type, PD-L1 expression, CT regimen, and cycles of nICT. The stability of the pCR rate was assessed by sensitivity analysis. The presence of publication bias was evaluated using the funnel plot.

## Results

### Eligible studies

**Figure 1:**
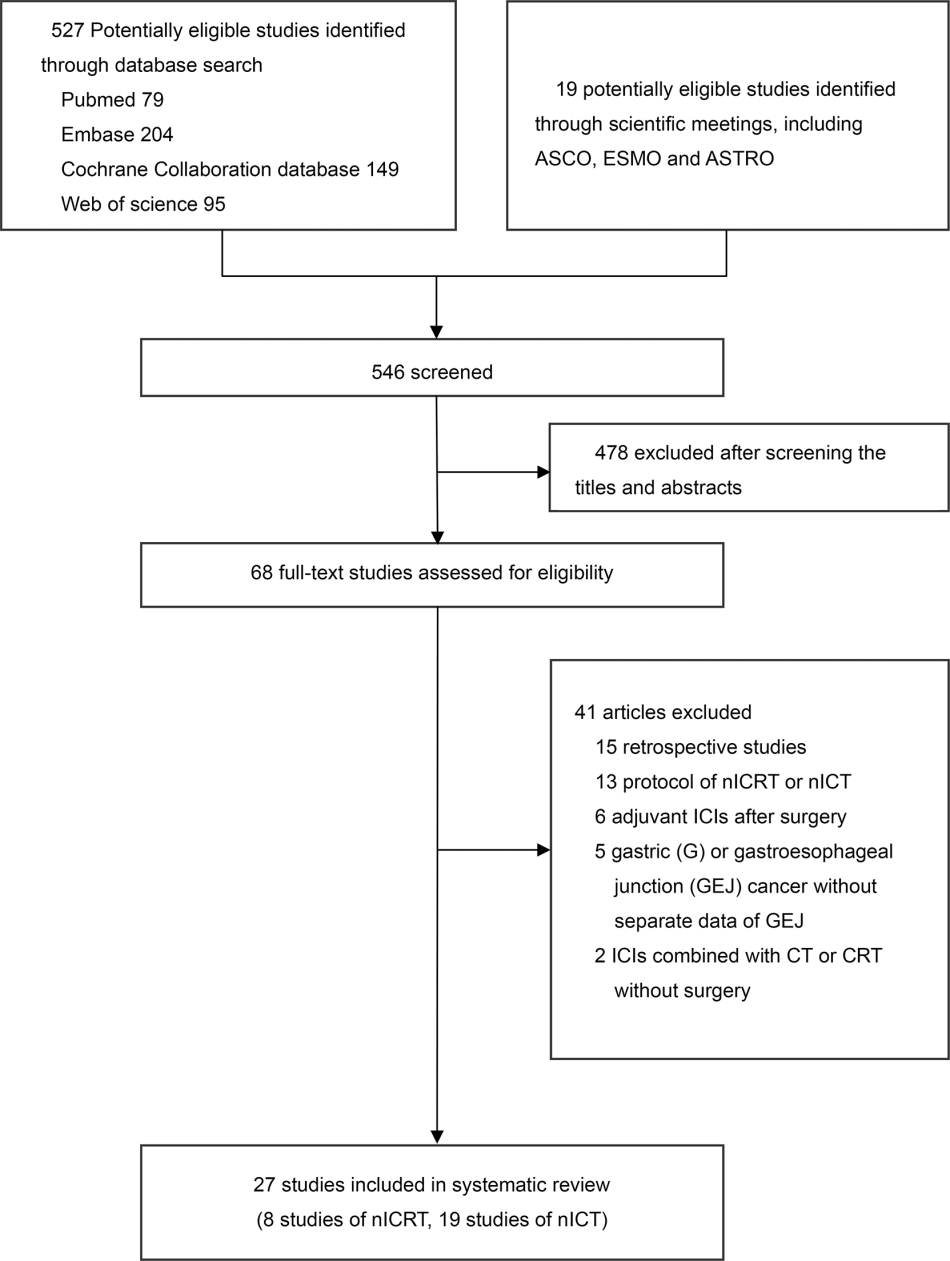
Literature search and selection. nICRT, neoadjuvant immune checkpoint inhibitor in combination with chemoradiotherapy; nICT, neoadjuvant immune checkpoint inhibitor in combination with chemotherapy.

The initial search retrieved 546 articles. After screening the abstract and/or full text, 519 articles were excluded. Finally, 27 studies were eligible for inclusion. The selection process and reasons for study exclusion are shown in **Figure 1**. Among the 27 studies, 8 trials with 221 patients examined nICRT (14–21), 19 trials with 588 patients examined nICT (22–40). Except one two-arms trial (33), all included studies were single-arm trials. Most of trials were conducted in China (20/27). ICI was concurrently administered with CT or CRT in all studies except one trial (33). The most commonly CT regimen was paclitaxel plus carboplatin or cisplatin (PC/TP) (18/27). The frequently adopted RT dose in studies of nICRT was 41.4Gy in 23 fractions (4/8). The median patient age were 63 years (interquartile range [IQR], 62-64 years) and 62 years (IQR, 61-64) for patients receiving nICRT and nICT, respectively; and the median sample sizes were 26 participants (IQR, 22-37) and 28 participants (IQR, 20-41), respectively. The main characteristics and outcomes of studies are presented in **Tables 1**, **2**.

**Table 1:** Characteristics of included trials.

First Author/Year	Country	Samplesize	male(%)	Medianage	Histological type	Clinicalstage	Intervention	CT regimen	Surgery type	Surgery time*	RT dose (Gy/f)	nICT cycle
Kelly/2019 (14)	USA	10	NR	NR	AC	2-3	Nivolumab+CRT	PC	MIE	6-10 w	NR	
Hong/2019 (15)	South Korea	28	NR	60	SCC	1b-3	Pembrolizumab+CRT	PC	NR	NR	44.1/21	
Ende/2021 (16)	Netherlands	40	88	63	AC	2-4a	Atezolizumab+CRT	PC	MIE	1-3 w	41.4/23	
Shah/2021 (17)	USA	42	80	68	AC	2-4a	Pembrolizumab+CRT	PC	NR	NR	41.4/23	
Li/2021 (18)	China	20	95	62	SCC	2-4a	Pembrolizumab+CRT	PC	MIE/open	4-9 w	41.4/23	
Uboha/2022 (19)	USA	22	91	64	SCC/AC	2-3	Avelumab+CRT	PC	NR	8-10 w	41.4/23	
Jiang/2022 (20)	China	23	NR	NR	SCC	1-4a	Toripalimab+CRT	PC	NR	4-6 w	30.0/12	
Cowzer/2022 (21)	USA	36	83	63	AC	2-4a	Durvalumab+CRT	FOLFOX	NR	6-8 w	50.4/28	
Zhang/2020 (22)	China	24	NR	NR	SCC	1-3	Toripalimab+CT	T+S-1	NR	NR		2-4
Alcindor/2021 (23)	Canada	28	89	NR	AC	2-4a	Avelumab+CT	DCF	NR	NR		4
Yang/2021 (24)	China	16	88	61	SCC	2-4a	Camrelizumab+CT	PC	MIE/open	4 w		2
Ma/2021 (25)	China	48	NR	62	SCC	NR	Camrelizumab+CT	TN	NR	NR		2-4
Shen/2021 (26)	China	28	96	62	SCC	2-4a	Anti-PD-1+CT	PC	MIE/open	3-5 w		2
Jiang/2021 (27)	China	42	NR	NR	SCC	2-3	Pembrolizumab+CT	TP	MIE	4-6 w		3
Li/2021 (28)	China	20	NR	NR	AC	3-4a	Sintilimab+CT	FLOT	D2	NR		3
Zhang/2021 (29)	China	30	87	58	SCC	3-4a	Sintilimab+CT	TP	MIE	4–6 w		2
Duan/2021 (30)	China	23	91	64	SCC	2-4a	Sintilimab+CT	P-based	MIE	4–6 w		3
Zhang/2021 (31)	China	40	NR	NR	SCC	2-4a	Sintilimab+CT	PC	NR	NR		2
Li/2021 (32)	China	17	NR	NR	AC	1-4a	Toripalimab+CT	FLOT	NR	NR		4
Xing/2021 (33)	China	30	73	63	SCC	2-4a	Toripalimab+CT	TP	MIE/open	4–6 w		2
Yan/2021 (34)	China	45	60	64	SCC	2-4a	Tislelizumab+CT	PC	MIE	3-6 w		3
Liu/2022 (35)	China	60	83	65	SCC	3-4a	Camrelizumab+CT	PC	NR	NR		2
Liu/2022 (36)	China	56	75	61	SCC	2-4a	Camrelizumab+CT	TP	MIE	6 w		2
Yang/2022 (37)	China	23	96	59	SCC	2-3	Camrelizumab+CT	PC	MIE	3-6 w		2
He/2022 (38)	China	20	75	61	SCC	3-4a	Toripalimab+CT	PC	MIE	4-6 w		2
Gao/2022 (39)	China	20	85	58	SCC	2-4a	Toripalimab+CT	DP	MIE	4-6 w		2
Duan/2022 (40)	China	18	78	64	SCC	2-4a	Pembrolizumab+CT	P-based	MIE	4-6 w		3

nICT, immune checkpoint inhibitor in combination with chemotherapy; CT, chemotherapy; RT, radiotherapy; CRT, chemoradiotherapy; SCC, squamous cell carcinoma; AC, adenocarcinoma; PC, paclitaxel+carboplatin; T, paclitaxel; DCF, docetaxel+cisplatin+5-FU; TN, paclitaxel+nedaplatin; TP, paclitaxel+cisplatin; P-based, platinum-based; FLOT, docetaxel+oxaliplatin+5-FU; DP, docetaxel+cisplatin; MIE; McKeown or Ivor-Lewis esophagectomy; w, week; NR, not reported; f, fraction.

*Surgery time: time interval between surgery and the last dose of neoadjuvant therapy.

**Table 2:** Main outcomes of included trials.

First Author/Year		Intervention	pCR	Surgical resection	R0resection	Surgical delay	surgicalmortality	Grade ≥3TRAEs
Kelly/2019 (14)		Nivolumab+CRT	40%	NR	NR	0%	0%	NR
Hong/2019 (15)		Pembrolizumab+CRT	NR	93%	NR	NR	8%	NR
Ende/2021 (16)		Atezolizumab+CRT	30%	83%	100%	0%	0%	43%
Shah/2021 (17)		Pembrolizumab+CRT	13%	93%	NR	NR	NR	NR
Li/2021 (18)		Pembrolizumab+CRT	56%	90%	94%	0%	NR	65%
Uboha/2022 (19)		Avelumab+CRT	26%	86%	79%	NR	NR	NR
Jiang/2022 (20)		Toripalimab+CRT	55%	87%	NR	NR	NR	NR
Cowzer/2022 (21)		Durvalumab+CRT	24%	92%	NR	NR	NR	NR
Zhang/2020 (22)		Toripalimab+CT	17%	NR	NR	NR	NR	NR
Alcindor/2021 (23)		Avelumab+CT	11%	96%	96%	NR	0%	NR
Yang/2021 (24)		Camrelizumab+CT	31%	100%	94%	NR	NR	NR
Ma/2021 (25)		Camrelizumab+CT	35%	NR	NR	NR	NR	NR
Shen/2021 (26)		Anti-PD-1+CT	33%	96%	96%	NR	0%	7%
Jiang/2021 (27)		Pembrolizumab+CT	41%	NR	100%	0%	NR	0%
Li/2021 (28)		Sintilimab+CT	NR	85%	88%	0%	NR	NR
Zhang/2021 (29)		Sintilimab+CT	17%	77%	100%	0%	0%	3%
Duan/2021 (30)		Sintilimab+CT	35%	74%	94%	0%	0%	30%
Zhang/2021 (31)		Sintilimab+CT	25%	100%	98%	0%	NR	NR
Li/2021 (32)		Toripalimab+CT	33%	88%	100%	NR	NR	NR
Xing/2021 (33)		Toripalimab+CT	21%	80%	100%	NR	4%	NR
Yan/2021 (34)		Tislelizumab+CT	44%	80%	81%	0%	3%	33%
Liu/2022 (35)		Camrelizumab+CT	39%	85%	98%	16%	0%	57%
Liu/2022 (36)		Camrelizumab+CT	31%	91%	100%	NR	0%	11%
Yang/2022 (37)		Camrelizumab+CT	25%	87%	100%	0%	0%	NR
He/2022 (38)		Toripalimab+CT	19%	80%	88%	0%	NR	20%
Gao/2022 (39)		Toripalimab+CT	0%	60%	100%	0%	0%	NR
Duan/2022 (40)	Pembrolizumab+CT		31%	72%	85%	0%	8%	28%

CT, chemotherapy; CRT, chemoradiotherapy; pCR, pathological complete response; TRAEs, treatment-related adverse events; NR, not reported.

### Assessment of included studies and publication bias

There was only one two-arms trial (33) which was rated with a unclear risk of bias, others were single-arm trials. As single-arm trials have a high risk of bias by their nature, they were not further assessed for bias. The funnel plots for pCR rate suggested a probability of publication bias, especially for nICT (**Supplementary File: Figure S1**).

### pCR rate

**Figure 2:**
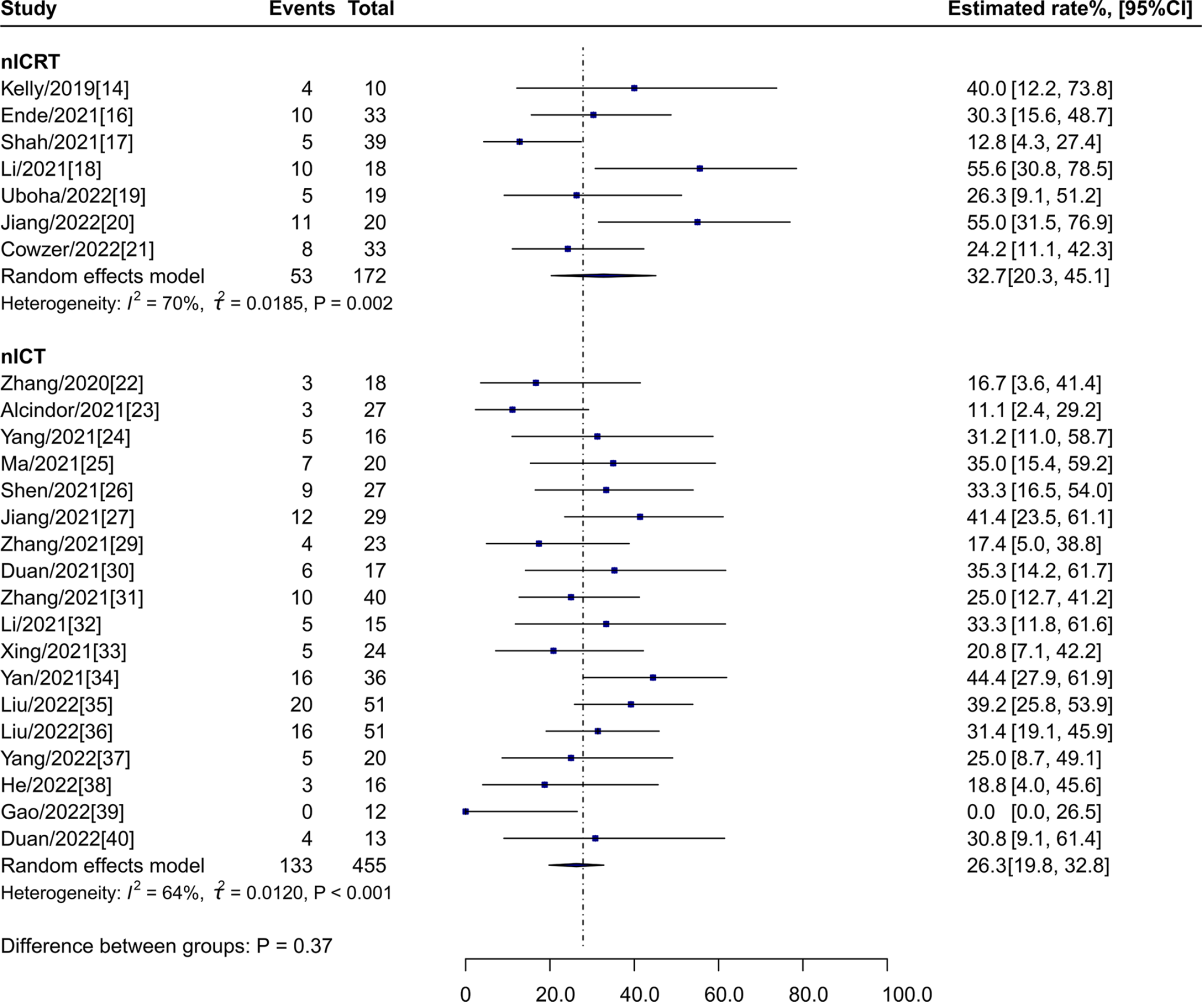
pCR rates of nICRT vs nICT. pCR, pathological complete response; nICRT, neoadjuvant immune checkpoint inhibitor in combination with chemoradiotherapy; nICT, neoadjuvant immune checkpoint inhibitor in combination with chemotherapy.

The estimated pCR rate for nICRT was 32.7% (95% CI: 20.3%-45.1%, I^2^ = 71%) vs 26.3% (95% CI: 19.8%-32.8%, I^2^ = 64%; P = 0.37) for nICT (**Figure 2**).

### Surgical safety

**Figure 3:**
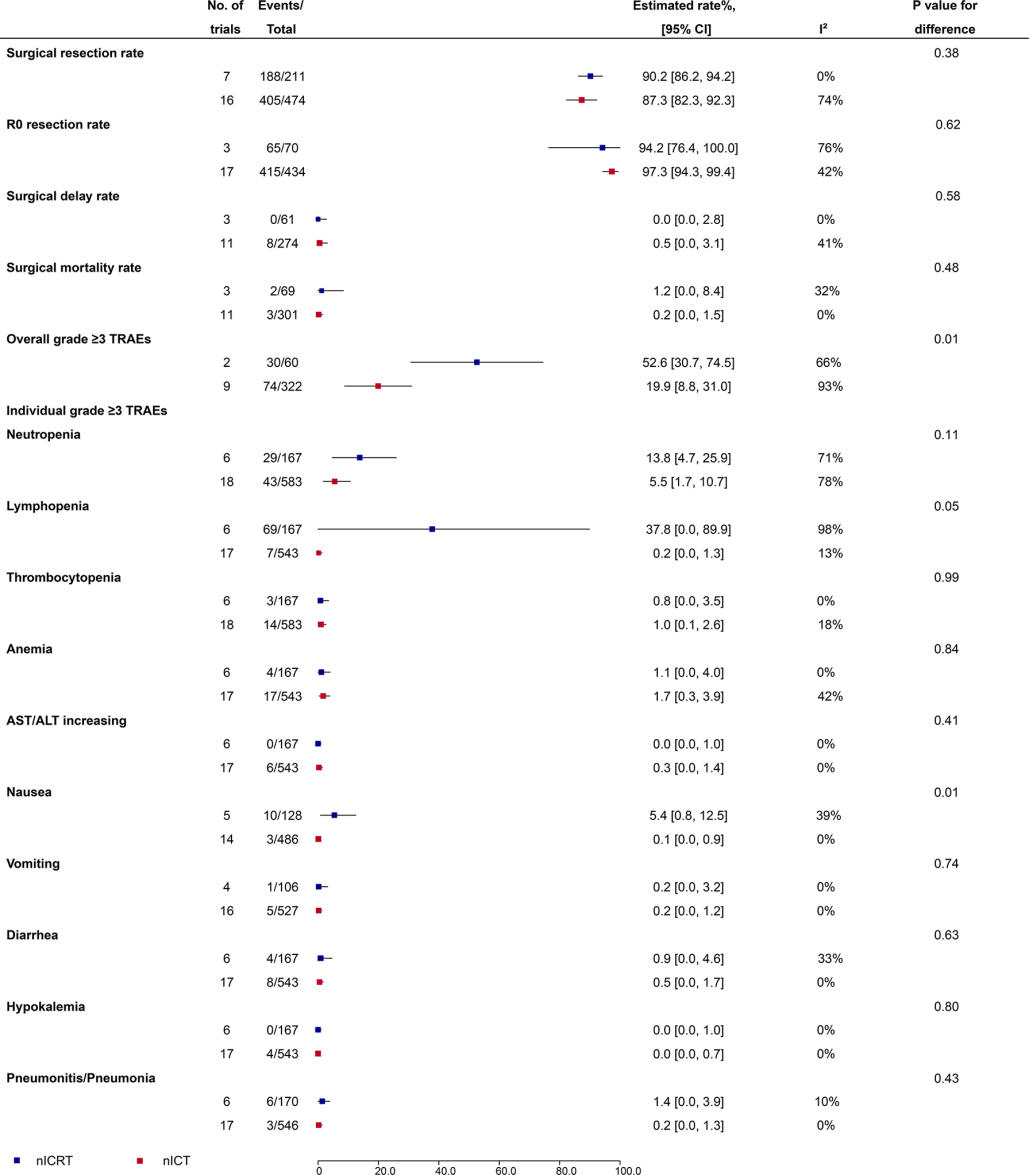
Surgical safety and grade ≥3 TRAEs of nICRT vs nICT. TRAEs, treatment-related adverse events; nICRT, neoadjuvant immune checkpoint inhibitor in combination with chemoradiotherapy; nICT, neoadjuvant immune checkpoint inhibitor in combination with chemotherapy.

Surgical resection rate (90.2%, 95% CI: 86.2%-94.2%, I^2^ = 0% vs 87.3%, 95% CI: 82.3%-92.3%, I^2^ = 74%; P = 0.38), R0 resection rate (94.2%, 95% CI: 76.4%-100%, I^2^ = 76% vs 97.3%, 95% CI: 94.3%-99.4%, I^2^ = 42%; P = 0.62), surgical delay rate (0.0%, 95% CI: 0.0%-2.8%, I^2^ = 0% vs 0.5%, 95% CI: 0.0%-3.1%, I^2^ = 41%; P = 0.58), and surgical mortality rate (1.2%, 95% CI: 0.0%-8.4%, I^2^ = 32% vs 0.2%, 95% CI: 0.0%-1.5%, I^2^ = 0%; P = 0.48) were comparable between nICRT and nICT (**Figure 3**).

### Incidence of grade ≥3 TRAEs

Incidence of the overall grade ≥3 TRAEs was significantly higher in patients receiving nICRT compared to patients receiving nICT (52.6%, 95% CI: 30.7%-74.5%, I^2^ = 66% vs 19.9%, 95% CI: 8.8%-31.0%, I^2^ = 93%; P = 0.01) (**Figure 3**). Further analyses of individual grade ≥3 TRAEs (**Figure 3**) showed that nICRT was associated with more lymphopenia (37.8%, 95% CI: 0.0%-89.9%, I^2^ = 98% vs 0.2%, 95% CI: 0.0%-1.3%, I^2^ = 13%; P = 0.05) and nausea (5.4%, 95% CI: 0.8%-12.5%, I^2^ = 39% vs 0.1%, 95% CI: 0.0%-0.9%, I^2^ = 0%; P = 0.01) than nICT; other individual grade ≥3 TRAEs including neutropenia, thrombocytopenia, anemia, AST/ALT increasing, vomiting, diarrhea, hypokalemia, and pneumonitis/pneumonia were comparable between nICRT and nICT (P > 0.05 for each comparison).

### Subgroup analysis

**Figure 4:**
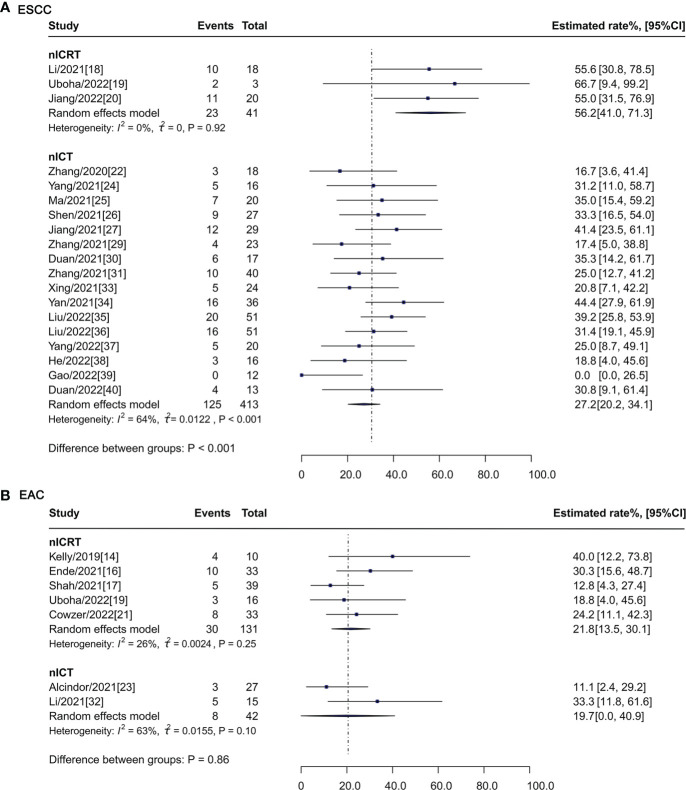
Subgroup analysis of pCR rate according to histological type. pCR, pathological complete response; nICRT, neoadjuvant immune checkpoint inhibitor in combination with chemoradiotherapy; nICT, neoadjuvant immune checkpoint inhibitor in combination with chemotherapy; ESCC, esophageal squamous cell carcinoma; EAC, esophageal adenocarcinoma.

For patients with ESCC (**Figure 4A**), pCR rate of nICRT was 56.2% (95% CI: 41.0%-71.3%, I^2^ = 0%), which was significantly higher than that of nICT (27.2%, 95% CI: 20.2%-34.1%, I^2^ = 64%; P < 0.001). pCR rates were similar between nICRT (21.8%, 95% CI: 13.5%-30.1%, I^2^ = 26%) and nICT (19.7%, 95% CI: 0.0%-40.9%, I^2^ = 63%; P = 0.86) in patients with EAC (**Figure 4B**).

**Figure 5:**
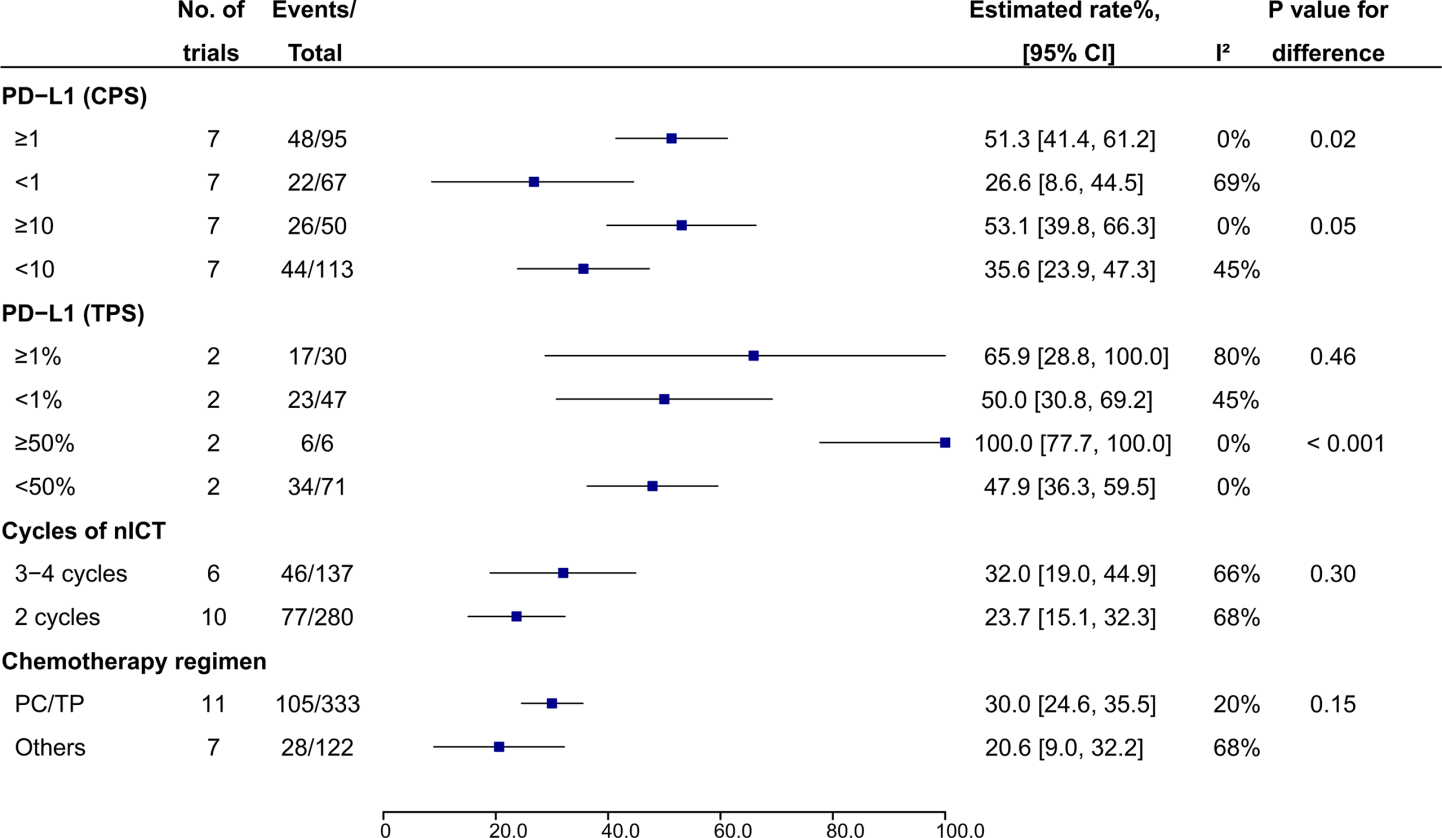
Subgroup analysis of pCR rate in patients receiving nICT. nICT, neoadjuvant immune checkpoint inhibitor in combination with chemotherapy; PC/TP, paclitaxel plus carboplatin or cisplatin.

In patients treated with nICT, PD-L1 expression Combined Positive Score (CPS) ≥1 (51.3%, 95% CI: 41.4%-61.2%, I^2^ = 0%) and CPS ≥10 (53.1%, 95% CI: 39.8%-66.3%, I^2^ = 0%) were associated higher pCR rates compared to CPS <1 (26.6%, 95% CI: 8.6%-44.5%, I^2^ = 69%; P = 0.02) and CPS <10 (35.6%, 95% CI: 23.9%-47.3%, I^2^ = 45%; P = 0.05), respectively (**Figure 5**). PD-L1 expression Tumor Proportion Score (TPS) ≥ 50% also had a higher pCR rate compared to those with TPS <50% (100.0%, 95% CI: 77.7%-100.0%, I^2^ = 0% vs 47.9%, 95% CI: 36.3%-59.5%, I^2^ = 0%; P < 0.001), while no difference was observed between TPS ≥1% and <1% (65.9%, 95% CI: 28.8%-100%, I^2^ = 80% vs 50.0%, 95% CI: 30.8%-69.2%, I^2^ = 45%; P = 0.46) (**Figure 5**). With regard to CT regimen, PC/TP regimen showed a numerically higher pCR rate compared to the other regimens, while no significant statistical difference was observed (30.0%, 95% CI: 24.6%-35.5%; I^2^ = 20% vs 20.6%, 95% CI: 9.0%-32.2%, I^2^ = 68%; P = 0.15) (**Figure 5**); PC/TP regimen was also associated a numerically lower incidence of the overall grade ≥3 TRAEs (17.8%, 95% CI: 5.7%-29.9%; I^2^ = 94% vs 29.2%, 95% CI: 15.3%-43.2%; I^2^ = 0%; P = 0.22), while individual grade ≥3 TRAE was similar between the two groups (**Supplementary File: Figure S2**). In term of the number of cycles of nICT before surgery, pCR rates were comparable between 3-4 and 2 cycles (32.0%, 95% CI: 19.0%-44.9%, I^2^ = 66% vs 23.7%, 95% CI: 15.1%-32.3%, I^2^ = 68%; P = 0.30) (**Figure 5**); the overall and all individual grade ≥3 TRAEs between the two groups were also similar (**Supplementary File: Figure S3**).

### Sensitivity analysis

When individual trials of nICRT or nICT were removed one at a time from the analyses for pCR rate, the results were not markedly altered by any single study, indicating a good stability of these results (**Supplementary File: Figure S4**).

### Outcomes for nCRT and nCT

We also searched PubMed for publications of nCRT and nCT in EC after January 1, 2010. Finally, 13 trials of nCRT and 16 trials of nCT were eligible. The estimated pCR rate was 28.9 (95% CI: 24.2%-33.6%, I^2^ = 69%) for nCRT vs 10.3 (95% CI: 5.9%-14.6%, I^2^ = 85%) for nCT (P < 0.001). Surgical resection rate, R0 resection rate, and surgical mortality rate for nCRT and nCT appeared to be comparable between nCRT and nCT. The detailed results are shown in **Supplementary File: Figure S5**.

## Discussion

In theory, either RT or CT is able to improve the immunogenicity of the tumor microenvironment (45), and addition of ICI to RT/CT should be correlated with increased antitumor activity. However, which is the better choice for nICRT or nICT is still unclear. This is a comprehensive systematic review and meta-analysis assessing antitumor efficacy and safety of nICRT and nICT in patients with resectable EC. It showed that the estimated pCR rates for nICRT and nICT were 32.7% and 26.3% (P = 0.37), respectively, which were much higher than that for conventional nCT (10.3%, **Figure S5**) and appeared to be similar to that for conventional nCRT (28.9%, **Figure S5**). There were no significant differences in surgical resection rate, R0 resection rate, surgical delay rate, and surgical mortality rate between nICRT and nICT. However, nICRT was associated with a higher incidence of grade ≥3 TRAEs compared to nICT. Nevertheless, the increased grade ≥3 TRAEs in patients receiving nICRT were likely to be acceptable because further analyses of individual grade ≥3 TRAEs showed that only lymphopenia and nausea significantly increased in patients receiving nICRT without grade 5 TRAEs, which appeared to be manageable clinically; other individual grade ≥3 TRAEs seemed to be comparable between patients receiving nICRT and nICT.

Despite the estimated pCR rates being comparable between nICRT and nICT, results from individual studies are various (13%-55% for nICRT and 0%-44% for nICT), indicating that some factors may affect the antitumor activity of treatments. Histology is important in EC, with ESCC being more likely to have local recurrence and more sensitive to RT compared to EAC. In addition, ESCC may also be more sensitive to ICI than EAC due to having a relatively higher prevalence of high TMB or high PD-L1 expression (46, 47). Thus, patients with ESCC should be more benefit from nICRT theoretically. In our study, nICRT achieved a significantly higher pCR rate compared to nICT for ESCC (56.2% vs 27.2%, P < 0.001), but not for EAC (21.8 vs 19.7%, P = 0.86), supporting the first choice of nICRT for patients with ESCC. On the other hand, nICT might be taken into consideration in patients with EAC, particularly in elderly patients or those with poor performance, due to its similar antitumor efficacy and relatively low incidence of grade ≥3 TRAEs compared to nICRT.

PD-L1 expression has been demonstrated to be a potential biomarker for ICI efficacy in metastatic EC, while its predictive value for tumor pathologic response in neoadjuvant immunotherapy setting is under evaluation. Two trials of nICT (34, 37) showed that higher PD-L1 expression was associated higher rate of pCR in resectable EC. While, no predictive value of PD-L1 expression was observed in another two trials of nICT (30, 35). One possible explanation for the inconsistent findings is the small sample size of the trials, which is underpowered to detect the correlation between PD-L1 expression and pCR. In addition, difference in score system of PD-L1 expression (TPS or CPS) and cutoff point used in individual trials may also affect judgement of the results. It had been reported that TPS had a better prediction performance than CPS in EC (21). Moreover, PD-L1 expression can be upregulated by CT/CRT in patients with EC (38, 48, 49). In a phase 2 trial of EC (38), there were 5 patients (29%) whose CPS changed from 0 before nICT to ≥ 1 after nICT. Kelly et al. found that PD-L1 level was 45.2% and 77.4% before and after nCRT (48). These results suggest that some patients can benefit from nICRT or nICT independent of the prior PD-L1 status. In our subgroup analysis of nICT, the estimated pCR rates for patients with CPS ≥1 and CPS ≥10 were higher than those with PD-L1 expression CPS<1 (51.3% vs 26.6%, P = 0.02) and CPS<10 (53.1% vs 35.6%, P = 0.05), respectively. PD-L1 expression TPS ≥50% was associated a higher pCR rate than TPS<50%. Despite the positive findings, identifying PD-L1 expression as a predictor of pCR in patients with nICT needs more clinical data. In addition, its predictive role in patients receiving nICRT also needs to be further explored.

FP and PC are two common CT regimens used in conventional nCRT and nCT. While in current trials of nICRT or nICT, PC/TP was frequently adopted. In our study, although PC/TP regimen showed a numerically higher pCR rate (30.0% vs 20.6%) and a numerically lower incidence of grade ≥3 TRAEs (17.8% vs 29.2%) compared to other regimens in patients receiving nICT, significant statistical differences were not observed. Thus, it is still difficult to draw a conclusion on the superiority of PC/TP regimens over others. We also assessed the effect of cycles neoadjuvant treatment on the antitumor activity and safety of nICT. We found that 3-4 cycles of nICT was not associated with a significant improvement in pCR rate compared to 2 cycles of nICT (32.0% vs 23.7%, P = 0.30), despite without increased grade ≥3 TRAEs. These results support the use of two cycles of nICT before surgery, and extending the cycle to 3-4 cycles appears to have no additional benefits. However, it should be noted that almost all of the data of CT regimens and cycles of nICT are from patients with ESCC, whether the findings can extending to patients with EAC needs further evaluation.

ICI administering concurrently with CT/CRT is a common strategy in current trials. However, whether it is the optimal treatment modality is controversial. Results from a phase 2 trial of nICT in resectable EC (33) showed that toripalimab administered on day 3 resulted in a higher pCR rate than that administered on the same day with CT. Another retrospective study of lung cancer showed that administering ICIs 3-5 days after CT is superior to administering ICIs before or concurrent with CT (50). One possible reason for the results is that ICI can result in the expansion of tumor-specific T cells (51), and administering ICI after CT may decrease possibility of the proliferating T cells killed by CT drugs. Another explanation is that the upfront CT can lead to an upregulation of PD-L1 expression as mentioned above, which may improve antitumor activity of sequential ICI.

The optimal time for surgical resection after neoadjuvant immunotherapy is also yet to be determined. By two mouse tumor models, Liu et al. demonstrated that a short duration (4–5 days) between the first administration of neoadjuvant ICI and surgery was associated with better efficacy compared to extending the duration to 10 days (52). However, this short time point of surgery has never been examined in clinical trials of EC. In trials included in our study, surgery is generally performed 3-6 weeks after the last cycle neoadjuvant ICI.

In our meta-analysis, 41.4Gy in 23 fractions is a frequently used RT schedule in studies of nICRT. There are also trials adopted schedule of 44.1Gy in 21 fractions (15) or 50.4Gy in 28 fractions (21). Limited by insufficient number of studies, we could not compare the difference in pCR rate and safety between different dose schedules. However, in a recent systematic review of 110 studies with 7,577 EC patients treated with nCRT (53), patients receiving a dose of 40.0-41.4 Gy/20-23 fractions showed improved OS compared to those receiving above this dose. The findings support 40.0-41.4 Gy/20-23 fractions as a better RT strategy for patients receiving nCRT.

Despite promising pCR rate achieved either by nICRT or nICT, OS is the gold standard efficacy endpoint. Several trials reported 1-year (ranging from 77% to 100%) and/or 2-year (ranging from 53.7% to 85%) OS rates (**Supplementary File: Table S3**). However, to date, no trials have provided the long-term survival outcomes. Long-term follow-up is necessary to determine the correlation between pCR and long-term survival.

There are several limitations in the current study. First, almost all studies included this meta-analysis are single-arm trials, and the findings are hypothesis-generating. Lack of large head to head RCTs prevents us from making a firm conclusion. Second, number of studies and sample sizes were relatively small, especially in some subgroups such as subgroup of PD-L1 expression (TPS) for nICT and subgroups of surgical safety and grade ≥3 TRAEs for nICRT, which might lead to the results less reliable. Thus, these findings need to be validated in large-sample trials. Third, most of trials of nICT were performed in China, and thus, extending the conclusions to other regions should be discreet. Fourth, there are substantial heterogeneity among studies. By subgroup analyses, we found that histological subtypes, PD-L1 expression and CT regimens might account for some heterogeneity. For example, heterogeneity I^2^ was 70% for pCR in patients receiving nICRT, while I^2^ reduced to 0% in subgroup of ESCC; I^2^ was 63% for pCR in patients receiving nICT, while I^2^ decreased to 0% in subgroup of PD-L1 CPS ≥1 or ≥10 and decreased to 20% in CT regimen with PC/TP. In addition, type of ICI (PD-1 or PD-L1 inhibitor or different agents of the same type), tumor location (Upper or lower or esophagogastric junction), and clinical stage (1-2 or 3-4a stage) may also confounding factors. However, we could not analyze their effects on pCR due to limited data or studies. Finally, some conference abstracts included in our study did not provided full information of surgical safety and/or TRAEs, which could result in a selection bias because these data might be reported in the full publication.

## Conclusion

Both nICT and nICRT achieved promising pCR rates with acceptable tolerability, and nICRT was likely to have more antitumor efficacy compared to nICT for patients with SCC. PD-L1 status seemed to be predictive of pCR in patients receiving nICT; CT with PC/TP regimen and increasing cycles of nICT did not appear to significantly improve pCR rate. Nevertheless, these findings are hypothesis generating and require further validation by large RCTs. Moreover, future trials of nICI with long-term survival outcomes are wanted to clarify the correlation between pCR and OS.

## Data availability statement

The original contributions presented in the study are included in the article/**Supplementary Material**. Further inquiries can be directed to the corresponding author.

## Ethics statement

Ethical review and approval was not required for the study on human participants in accordance with the local legislation and institutional requirements. Written informed consent for participation was not required for this study in accordance with the national legislation and the institutional requirements.

## Author contributions

Conception and design: JD. Collection and assembly of data: HWand SL and TL. Data analysis and interpretation: All authors. Manuscript writing: All authors. Final approval of manuscript: All authors.

## Conflict of interest

The authors declare that the research was conducted in the absence of any commercial or financial relationships that could be construed as a potential conflict of interest.

## Publisher’s note

All claims expressed in this article are solely those of the authors and do not necessarily represent those of their affiliated organizations, or those of the publisher, the editors and the reviewers. Any product that may be evaluated in this article, or claim that may be made by its manufacturer, is not guaranteed or endorsed by the publisher.
