# Supplementary material for: Neoadjuvant immune checkpoint inhibitor in combination with chemotherapy or chemoradiotherapy in resectable esophageal cancer: A systematic review and meta-analysis
Source: Front Immunol. 2022 Sep 13;13:998620. doi: 10.3389/fimmu.2022.998620 (PMC9513123; doi:10.3389/fimmu.2022.998620)
Supplement: Supplementary file 1 [file DataSheet_1.doc]

**List of supplementary file：**

**Table S1** PRISMA Checklist (page 2-4)

**Table S2** Search strategy (page 5-7)

**Table S3** Survival outcomes

**Figure S1** Funnel plots of publication bias for pCR rate (page 8).

**Figure S2** Incidence of grade ≥3 TRAEs according to chemotherapy regimen (page 9).

**Figure S3** Incidence of grade ≥3 TRAEs according to cycles of nICT (page 10).

**Figure S4** Sensitivity analysis for pCR rate (page 11).

**Figure S5** pCR rate and surgical safety for patients receiving nCRT and nCT (page 12).

**List of studies excluded at full-text screening stage** (page 13-18)

**Table S1 PRISMA Checklist**

| **Section/Topic** | **Item #** | **Checklist Item** | **Reported on Page #** |
| --- | --- | --- | --- |
| **TITLE** |  |  |  |
| Title | 1 | Identify the report as a systematic review incorporating a meta-analysis (or related form of meta-analysis). | 1 |
| **ABSTRACT** |  |  |  |
| Structured summary | 2 | Provide a structured summary including, as applicable:  **Background:** main objectives  **Methods:** data sources; study eligibility criteria, participants, and interventions; study appraisal; and synthesis methods, such as network meta-analysis.  **Results:** number of studies and participants identified; summary estimates with corresponding confidence/credible intervals; treatment rankings may also be discussed. Authors may choose to summarize pairwise comparisons against a chosen treatment included in their analyses for brevity.  **Discussion/Conclusions:** limitations; conclusions and implications of findings.  **Other:** primary source of funding; systematic review registration number with registry name. | 2-3 |
| **INTRODUCTION** |  |  |  |
| Rationale | 3 | Describe the rationale for the review in the context of what is already known, including mention of why a network meta-analysis has been conducted. | 4 |
| Objectives | 4 | Provide an explicit statement of questions being addressed, with reference to participants, interventions, comparisons, outcomes, and study design (PICOS). | 4-5 |
| **METHODS** |  |  |  |
| Protocol and registration | 5 | Indicate whether a review protocol exists and if and where it can be accessed (e.g., Web address); and, if available, provide registration information, including registration number. | INPLASY202260052 |
| Eligibility criteria | 6 | Specify study characteristics (e.g., PICOS, length of follow-up) and report characteristics (e.g., years considered, language, publication status) used as criteria for eligibility, giving rationale. Clearly describe eligible treatments included in the treatment network, and note whether any have been clustered or merged into the same node (with justification). | 6 |
| Information sources | 7 | Describe all information sources (e.g., databases with dates of coverage, contact with study authors to identify additional studies) in the search and date last searched. | 5 |
| Search | 8 | Present full electronic search strategy for at least one database, including any limits used, such that it could be repeated. | Supplementary Table S2 |
| Study selection | 9 | State the process for selecting studies (i.e., screening, eligibility, included in systematic review, and, if applicable, included in the meta-analysis). | 5 |
| Data collection process | 10 | Describe method of data extraction from reports (e.g., piloted forms, independently, in duplicate) and any processes for obtaining and confirming data from investigators. | 6 |
| Data items | 11 | List and define all variables for which data were sought (e.g., PICOS, funding sources) and any assumptions and simplifications made. | 7 |
| **Geometry of the network** | **S1** | Describe methods used to explore the geometry of the treatment network under study and potential biases related to it. This should include how the evidence base has been graphically summarized for presentation, and what characteristics were compiled and used to describe the evidence base to readers. | NA |
| Risk of bias within individual studies | 12 | Describe methods used for assessing risk of bias of individual studies (including specification of whether this was done at the study or outcome level), and how this information is to be used in any data synthesis. | 6 |
| Summary measures | 13 | State the principal summary measures (e.g., risk ratio, difference in means). Also describe the use of additional summary measures assessed, such as treatment rankings and surface under the cumulative ranking curve (SUCRA) values, as well as modified approaches used to present summary findings from meta-analyses. | 7 |
| Planned methods of analysis | 14 | Describe the methods of handling data and combining results of studies for each network meta-analysis. This should include, but not be limited to:   - Handling of multi-arm trials; - Selection of variance structure; - Selection of prior distributions in Bayesian analyses; and - Assessment of model fit. | 7 |
| **Assessment of Inconsistency** | **S2** | Describe the statistical methods used to evaluate the agreement of direct and indirect evidence in the treatment network(s) studied. Describe efforts taken to address its presence when found. | NA |
| Risk of bias across studies | 15 | Specify any assessment of risk of bias that may affect the cumulative evidence (e.g., publication bias, selective reporting within studies). | 7 |
| Additional analyses | 16 | Describe methods of additional analyses if done, indicating which were pre-specified. This may include, but not be limited to, the following:   - Sensitivity or subgroup analyses; - Meta-regression analyses; - Alternative formulations of the treatment network; and - Use of alternative prior distributions for Bayesian analyses (if applicable). | 7 |
| **RESULTS** |  |  |  |
| Study selection | 17 | Give numbers of studies screened, assessed for eligibility, and included in the review, with reasons for exclusions at each stage, ideally with a flow diagram. | 7-8 |
| **Presentation of network structure** | **S3** | Provide a network graph of the included studies to enable visualization of the geometry of the treatment network. | NA |
| **Summary of network geometry** | **S4** | Provide a brief overview of characteristics of the treatment network. This may include commentary on the abundance of trials and randomized patients for the different interventions and pairwise comparisons in the network, gaps of evidence in the treatment network, and potential biases reflected by the network structure. | NA |
| Study characteristics | 18 | For each study, present characteristics for which data were extracted (e.g., study size, PICOS, follow-up period) and provide the citations. | Table 1 |
| Risk of bias within studies | 19 | Present data on risk of bias of each study and, if available, any outcome level assessment. | 8 |
| Results of individual studies | 20 | For all outcomes considered (benefits or harms), present, for each study: 1) simple summary data for each intervention group, and 2) effect estimates and confidence intervals. Modified approaches may be needed to deal with information from larger networks. | 8-11 |
| Synthesis of results | 21 | Present results of each meta-analysis done, including confidence/credible intervals. In larger networks, authors may focus on comparisons versus a particular comparator (e.g. placebo or standard care), with full findings presented in an appendix. League tables and forest plots may be considered to summarize pairwise comparisons. If additional summary measures were explored (such as treatment rankings), these should also be presented. | 8-11 |
| **Exploration for inconsistency** | **S5** | Describe results from investigations of inconsistency. This may include such information as measures of model fit to compare consistency and inconsistency models, P values from statistical tests, or summary of inconsistency estimates from different parts of the treatment network. | NA |
| Risk of bias across studies | 22 | Present results of any assessment of risk of bias across studies for the evidence base being studied. | 8 |
| Results of additional analyses | 23 | Give results of additional analyses, if done (e.g., sensitivity or subgroup analyses, meta-regression analyses, alternative network geometries studied, alternative choice of prior distributions for Bayesian analyses, and so forth). | 10-11 |
| **DISCUSSION** |  |  |  |
| Summary of evidence | 24 | Summarize the main findings, including the strength of evidence for each main outcome; consider their relevance to key groups (e.g., healthcare providers, users, and policy-makers). | 12-16 |
| Limitations | 25 | Discuss limitations at study and outcome level (e.g., risk of bias), and at review level (e.g., incomplete retrieval of identified research, reporting bias). Comment on the validity of the assumptions, such as transitivity and consistency. Comment on any concerns regarding network geometry (e.g., avoidance of certain comparisons). | 16-17 |
| Conclusions | 26 | Provide a general interpretation of the results in the context of other evidence, and implications for future research. | 17 |
| **FUNDING** |  |  |  |
| Funding | 27 | Describe sources of funding for the systematic review and other support (e.g., supply of data); role of funders for the systematic review. This should also include information regarding whether funding has been received from manufacturers of treatments in the network and/or whether some of the authors are content experts with professional conflicts of interest that could affect use of treatments in the network. | NA |

PICOS = population, intervention, comparators, outcomes, study design.

**Table S2** Search strategy

**a:** Search strategy in PubMed

| # | Query |
| --- | --- |
| #1 | “Esophageal Neoplasms”[mh] |
| #2 | Esophageal Neoplasm[tiab] OR Esophagus Neoplasm[tiab] OR Esophagus Cancer[tiab] OR Esophageal Cancer[tiab] OR Esophageal Cancers[tiab] |
| #3 | Esophageal[tiab] OR Oesophageal[tiab] OR Esophagus[tiab] OR Esophag*[tiab] OR Oesophag*[tiab] |
| #4 | Cancer*[tiab] OR Tumour*[tiab] OR Tumor[tiab] OR Neoplasm*[tiab] OR Carcinoma[tiab] |
| #5 | #3 AND #4 |
| #6 | #1 OR #2 OR #5 |
| #7 | "Esophagogastric Junction"[Mesh] |
| #8 | Junction, Esophagogastric[tiab] OR Gastroesophageal Junction[tiab] OR Gastroesophageal Junctions[tiab] OR Junction, Gastroesophageal[tiab] OR Junctions, Gastroesophageal[tiab] |
| #9 | #7 OR #8 |
| #10 | #9 AND #4 |
| #11 | #6 OR #10 |
| #12 | Neoadjuvant[tiab] OR Preoperative[tiab] OR Induction[tiab] |
| #13 | Chemoradiotherapy[tiab] OR Chemotherapy[tiab] OR Chemotherap*[tiab] OR Chemoradi*[tiab] OR Radiochemo*[tiab] OR Radiation therapy[tiab] OR Radiotherapy[tiab] |
| #14 | Immunotherapy[tiab] OR Immune checkpoint inhibitors[tiab] OR ICI[tiab] OR Programmed Death Ligand 1[tiab] OR PD-L1[tiab] OR Programmed Death 1[tiab] OR PD-1[tiab] OR Anti-Programmed Death Ligand 1[tiab] OR Anti-PD-L1[tiab] OR Anti-Programmed Death 1[tiab] OR Anti-PD-1[tiab] OR Atezolizumab[tiab] OR Durvalumab[tiab] OR Nivolumab[tiab] OR Pembrolizumab[tiab] OR Avelumab[tiab] OR Camrelizumab[tiab] OR Immunotherapy[tiab] OR Immune checkpoint inhibitors[tiab] OR ICI[tiab] OR Toripalimab[tiab] OR Sintilimab[tiab] OR Tislelizumab[tiab] |
| #15 | Randomized[pt] |
| #16 | Trial[pt] |
| #17 | Prospective[tiab] |
| #18 | #15 OR #16 OR #17 |
| #19 | #11 AND #12 AND #13 AND #14 AND #18 |

**b:** Search strategy in Embase

| # | Query |
| --- | --- |
| #1 | ‘esophagus tumor’/exp |
| #2 | ‘esophagus tumor’:ab,ti OR ‘esophageal neoplasm’:ab,ti OR ‘esophagus neoplasm’:ab,ti OR ‘esophagus cancer’:ab,ti OR ‘esophageal cancer’:ab,ti OR ‘esophageal cancers’:ab,ti OR ‘gastro-esophageal junction neoplasms’:ab,ti OR ‘esophagogastric junction neoplasms’:ab,ti |
| #3 | ‘esophageal’:ab,ti OR ‘oesophageal’:ab,ti OR ‘esophagus’:ab,ti OR ‘esophag*’:ab,ti OR ‘oesophag*’:ab,ti |
| #4 | ‘cancer*’:ab,ti OR ‘tumour*’:ab,ti OR ‘tumor’:ab,ti OR ‘neoplasm*’:ab,ti OR ‘carcinoma’:ab,ti |
| #5 | #3 AND #4 |
| #6 | #1 OR #2 OR #5 |
| #7 | ‘gastroesophageal junction’/exp |
| #8 | ‘junction, esophagogastric’:ab,ti OR ‘gastroesophageal junction’:ab,ti OR ‘gastroesophageal Junctions’:ab,ti OR ‘junction, gastroesophageal’:ab,ti OR ‘junctions, gastroesophageal’:ab,ti |
| #9 | #7 OR #8 |
| #10 | #9 AND #4 |
| #11 | #6 OR #10 |
| #12 | ‘neoadjuvant’:ab,ti OR ‘preoperative’:ab,ti OR ‘induction’:ab,ti |
| #13 | ‘chemoradiotherapy’:ab,ti OR ‘chemotherapy’:ab,ti OR ‘chemotherap*’:ab,ti OR ‘chemoradi*’:ab,ti OR ‘radiochemo*’:ab,ti OR ‘radiation therapy’:ab,ti OR ‘radiotherapy’:ab,ti |
| #14 | ‘programmed death ligand 1’:ab,ti OR ‘PD-L1’:ab,ti OR ‘programmed death 1’:ab,ti OR ‘PD-1’:ab,ti OR ‘anti-programmed death ligand 1’:ab,ti OR ‘anti-PD-L1’:ab,ti OR ‘anti-programmed death 1’:ab,ti OR ‘anti-PD-1’:ab,ti OR ‘atezolizumab’:ab,ti OR ‘durvalumab’:ab,ti OR ‘nivolumab’:ab,ti OR ‘pembrolizumab’:ab,ti OR ‘avelumab’:ab,ti OR ‘camrelizumab’:ab,ti OR ‘immunotherapy’:ab,ti OR ‘immune checkpoint inhibitors’:ab,ti OR ‘ICI’:ab,ti OR ‘toripalimab’:ab,ti OR ‘sintilimab’:ab,ti OR ‘tislelizumab’:ab,ti |
| #15 | 'prospective':ab,ti |
| #16 | 'trial':ab,ti |
| #17 | 'random*':ab,ti |
| #18 | #15 OR #16 OR #17 |
| #19 | #11 AND #12 AND #13 AND #14 AND #18 |

**c:** Search strategy in Cochrane Library

| # | Query |
| --- | --- |
| #1 | MeSH descriptor: [Esophageal Neoplasms] explode all trees |
| #2 | (esophageal neoplasm OR esophagus neoplasm OR esophagus cancer OR esophageal cancer OR esophageal cancers) |
| #3 | ((esophageal OR oesophageal OR esophagus OR esophag* OR oesophag*) AND (neoplas* OR cancer* OR carcinoma* OR tumour* or tumor)) |
| #4 | MeSH descriptor: [Esophagogastric Junction] explode all trees |
| #5 | (junction, esophagogastric OR gastroesophageal junction OR gastroesophageal junctions OR junction, gastroesophageal OR junctions, gastroesophageal) |
| #6 | (neoplas* OR cancer* OR carcinoma* OR tumour* or tumor) |
| #7 | #4 OR #5 |
| #8 | #7 AND #6 |
| #9 | #1 OR #2 OR #3 OR #8 |
| #10 | (neoadjuvant OR preoperative OR induction) |
| #11 | (chemoradiotherapy OR chemotherapy OR chemotherap* OR chemoradi* OR radiochemo* OR radiation therapy OR radiotherapy) |
| #12 | (Programmed Death Ligand 1 OR PD-L1 OR Programmed Death 1 OR PD-1 OR Anti-Programmed Death Ligand 1 OR Anti-PD-L1 OR Anti-Programmed Death 1 OR Anti-PD-1 OR Atezolizumab OR Durvalumab OR Nivolumab OR Pembrolizumab OR Avelumab OR Camrelizumab OR Immunotherapy OR Immune checkpoint inhibitors OR ICI OR Toripalimab OR Sintilimab OR Tislelizumab) |
| #13 | #9 AND #10 AND #11 AND #12 |

**d:** Search strategy in Web of Science

| # | Query |
| --- | --- |
| #1 | TS=("esophageal neoplasm” OR “esophagus neoplasm” OR “esophagus cancer” OR “esophageal cancer” OR “esophageal cancers” OR ((esophageal OR oesophageal OR esophagus OR esophag* OR oesophag*) AND (neoplas* OR cancer* OR carcinoma* OR tumour* or tumor))) |
| #2 | TS=(("esophagogastric junction” OR “junction, esophagogastric” OR “gastroesophageal junction” OR “gastroesophageal junctions” OR “junction, gastroesophageal” OR “junctions, jastroesophageal”) AND (neoplas* OR cancer* OR carcinoma* OR tumour* or tumor)) |
| #3 | #1 OR #2 |
| #4 | TS=(“neoadjuvant” OR “preoperative” OR “induction”) |
| #5 | TS=(“chemoradiotherapy” OR “chemotherapy” OR “chemotherap*” OR “chemoradi*” OR “radiochemo*” OR “radiation therapy” OR “radiotherapy”) |
| #6 | TS=(“programmed death ligand 1” OR “PD-L1” OR “programmed death 1” OR “PD-1” OR “anti-programmed death ligand 1” OR “anti-PD-L1” OR “anti-programmed death 1” OR “anti-PD-1” OR “atezolizumab” OR “durvalumab” OR “nivolumab” OR “pembrolizumab” OR “avelumab” OR “camrelizumab” OR “toripalimab” OR “sintilimab” OR “tislelizumab”) |
| #7 | TS=("randomized " OR "trial" OR "prospective") |
| #8 | #3 AND #4 AND #5 AND #6 AND #7 |

**Table S3** Survival outcomes

|  | 1-y OS | 1-y DFS | 1-y PFS | 2-y OS | 2-y DFS | 2-y PFS |
| --- | --- | --- | --- | --- | --- | --- |
| **nICRT** | | | | | | |
| Hong/2019[15] | 82.1% |  |  |  |  |  |
| Ende/2021[16] | 89.6% |  |  | 64.4% |  |  |
| Shah/2021[17] | 77.5% | 60.4% |  |  |  |  |
| Uboha/2022[19] | 77.0% | 67.0% |  |  |  |  |
| Cowzer/2022[21] | 92.0% | 82.0% | 81.0% | 85.0% | 78.0% | 78.0% |
| **nICT** | | | | | | |
| Yang/2021[24] | 90.9% |  | 83.0% |  |  |  |
| Liu/2022[36] | 100.0% |  | 92.4% |  |  |  |
| Yang/2022[37] |  | 79.8% |  |  |  |  |
| Duan/2022[41] | 88.3% |  |  | 53.7% |  |  |

nICRT, neoadjuvant immune checkpoint inhibitor in combination with chemoradiotherapy; nICT, neoadjuvant immune checkpoint inhibitor in combination with chemotherapy.


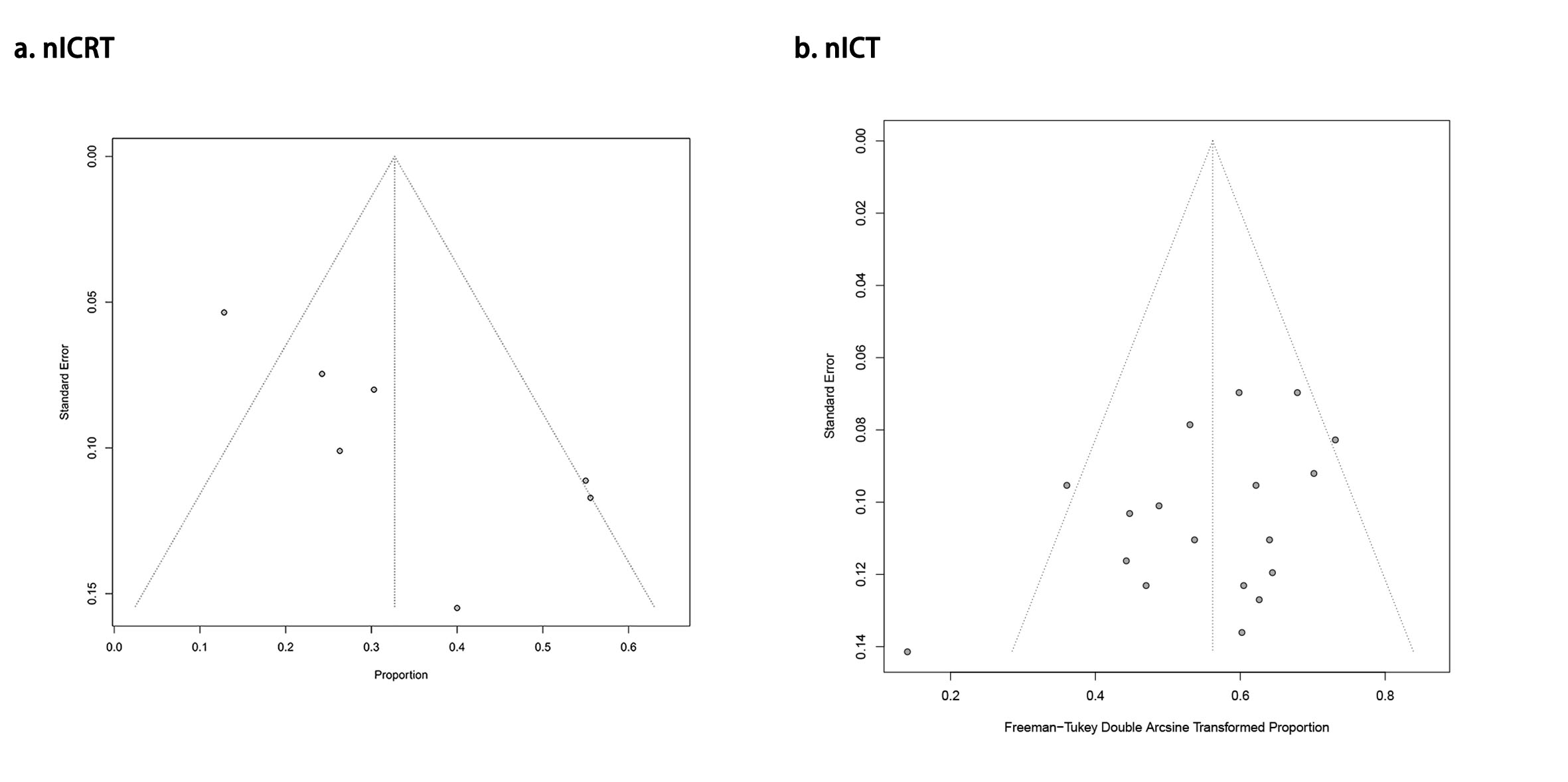


**Figure S1** Funnel plots of publication bias for pCR rate. pCR, pathological complete response; nICRT, neoadjuvant immune checkpoint inhibitor in combination with chemoradiotherapy; nICT, neoadjuvant immune checkpoint inhibitor in combination with chemotherapy.


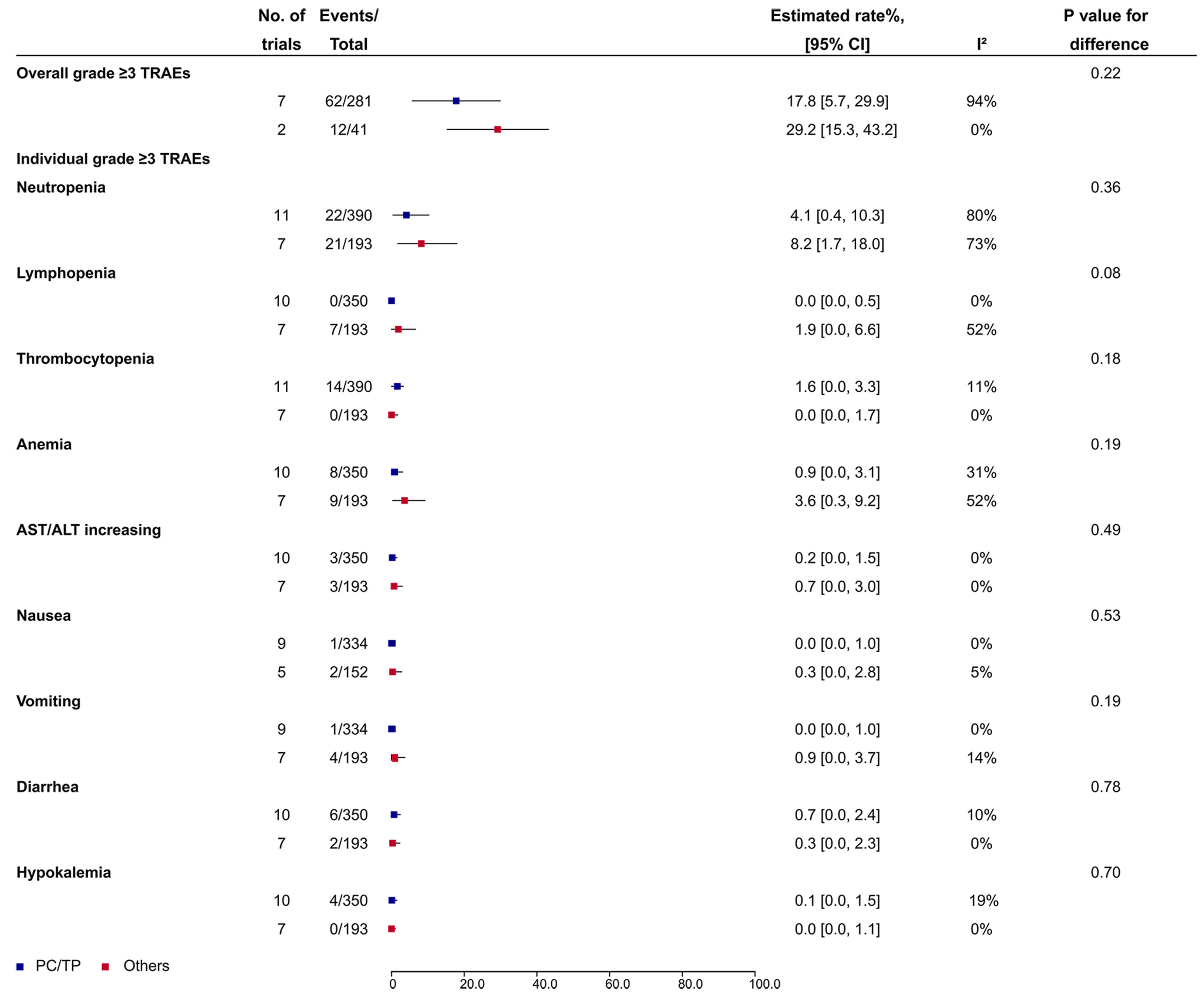


**Figure S2** Incidence of grade ≥3 TRAEs according to chemotherapy regimen. TRAEs, treatment-related adverse events; PC/TP, paclitaxel plus carboplatin or cisplatin.


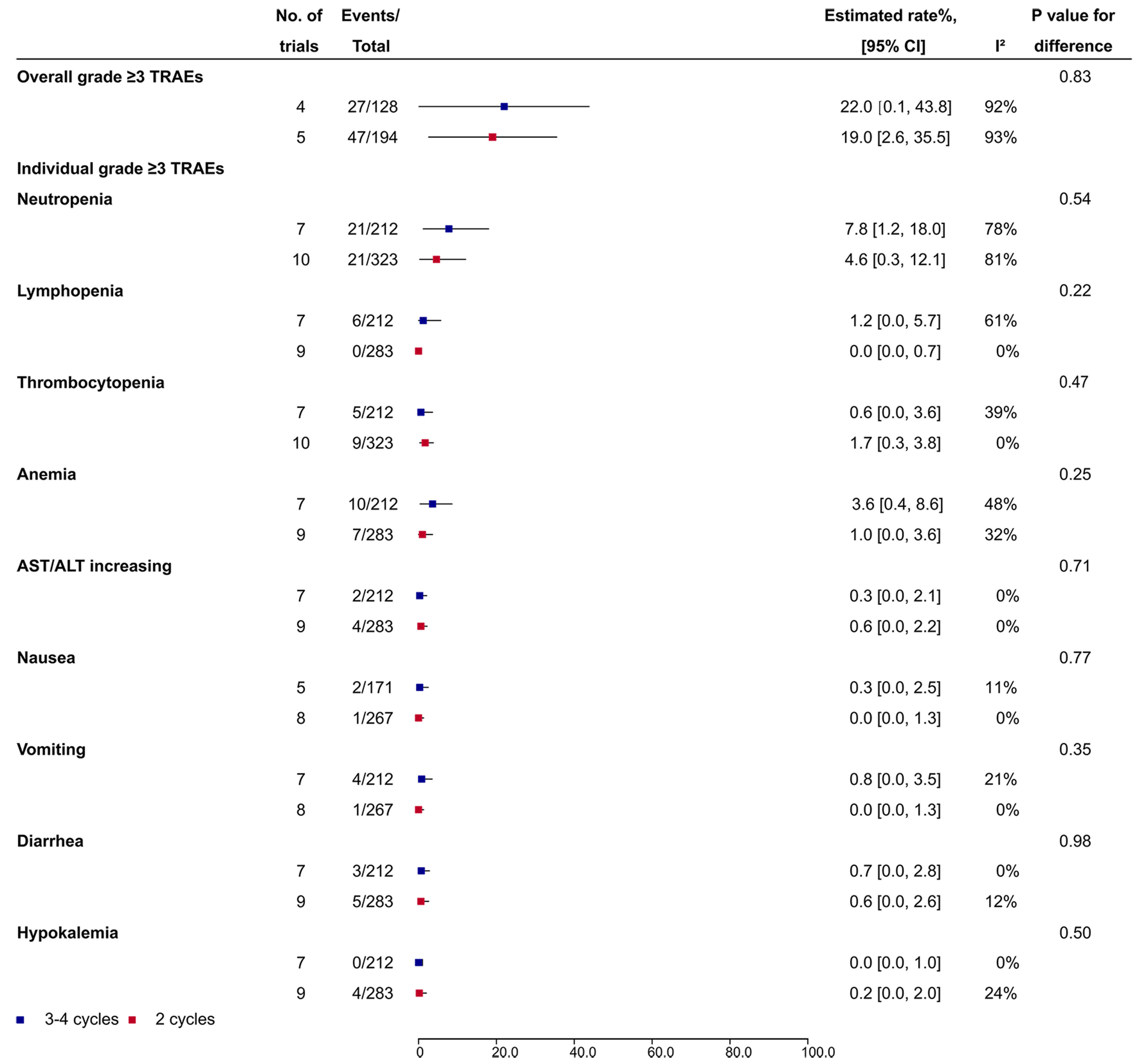


**Figure S3** Incidence of grade ≥3 TRAEs according to cycles of nICT. TRAEs, treatment-related adverse events; nICT, neoadjuvant immune checkpoint inhibitor in combination with chemotherapy.


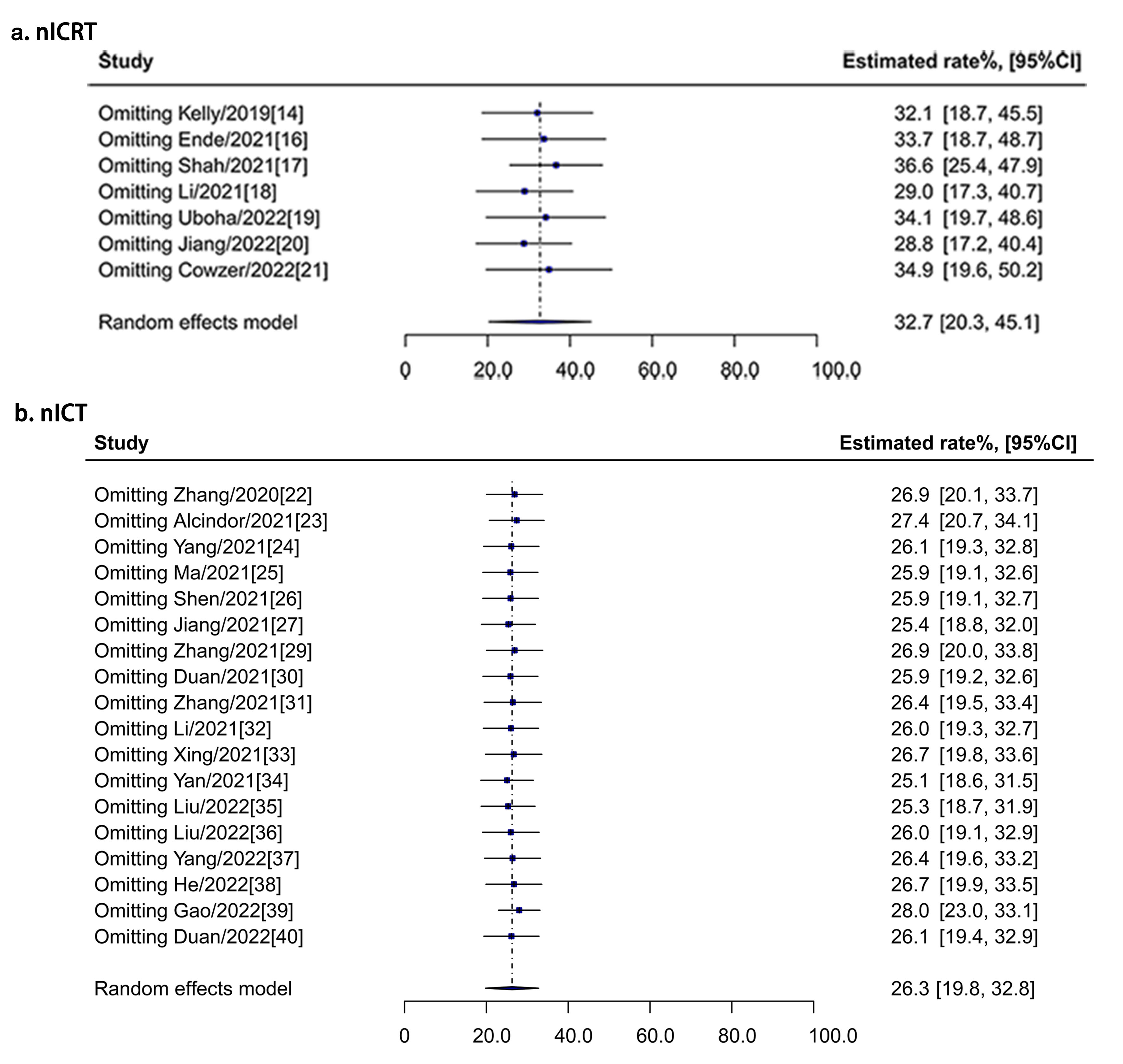


**Figure S4** Sensitivity analysis for pCR rate. pCR, pathological complete response; nICRT, neoadjuvant immune checkpoint inhibitor in combination with chemoradiotherapy; nICT, neoadjuvant immune checkpoint inhibitor in combination with chemotherapy.


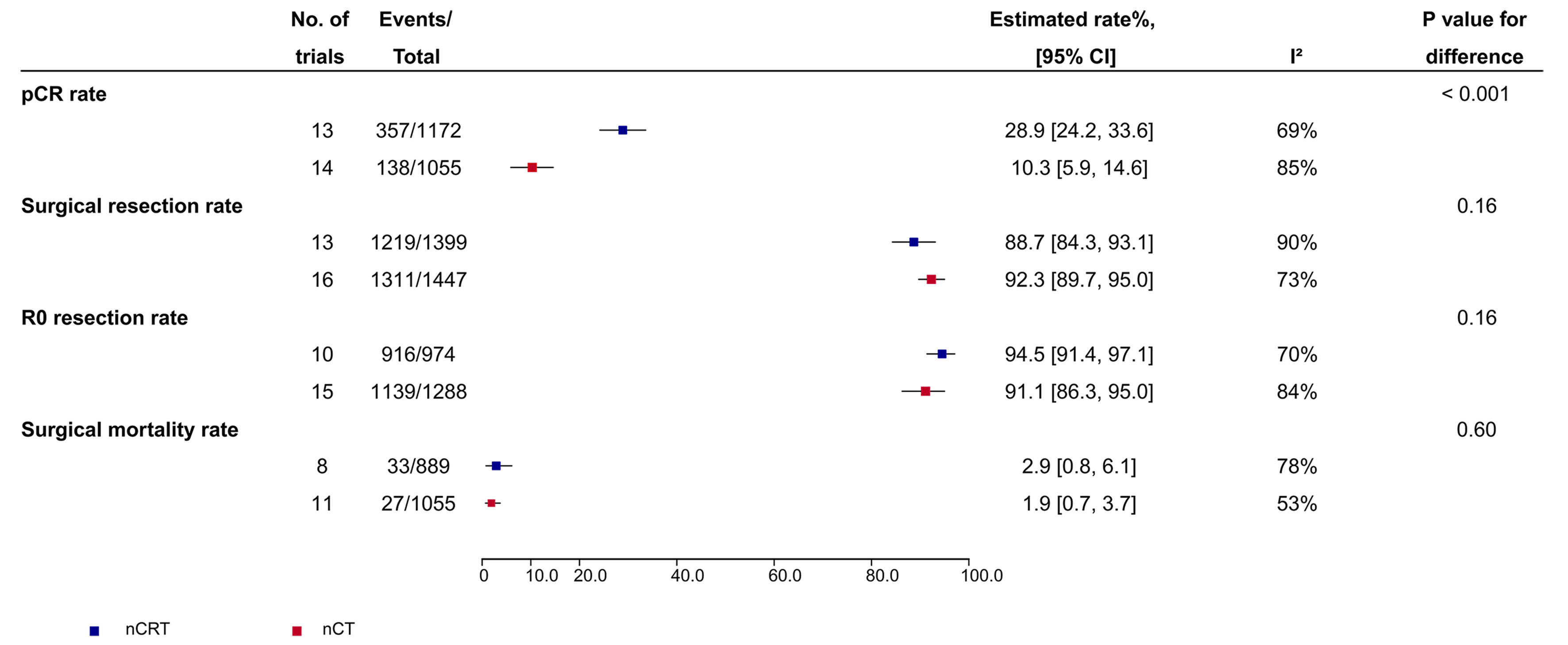


**Figure S5** pCR rate and surgical safety for patients receiving nCRT and nCT. pCR, pathological complete response; nCRT, neoadjuvant chemoradiotherapy; nCT, neoadjuvant chemotherapy.

**List of studies excluded at full-text screening stage：**

**Adjuvant ICIs after surgery (n=6)**

1. Park S, Sun JM, Choi YL, Oh D, Kim HK, Lee T, et al. Adjuvant durvalumab for esophageal squamous cell carcinoma after neoadjuvant chemoradiotherapy: a placebo-controlled, randomized, double-blind, phase II study. ESMO Open. 2022;7(1):100385.

2. Mamdani H, Schneider B, Perkins SM, Burney HN, Kasi PM, Abushahin LI, et al. A Phase II Trial of Adjuvant Durvalumab Following Trimodality Therapy for Locally Advanced Esophageal and Gastroesophageal Junction Adenocarcinoma: A Big Ten Cancer Research Consortium Study. Front Oncol. 2021;11:736620.

3. Smyth E, Knödler M, Giraut A, Mauer M, Nilsson M, Van Grieken N, et al. VESTIGE: Adjuvant Immunotherapy in Patients With Resected Esophageal, Gastroesophageal Junction and Gastric Cancer Following Preoperative Chemotherapy With High Risk for Recurrence (N+ and/or R1): An Open Label Randomized Controlled Phase-2-Study. Front Oncol. 2020;9:1320.

4. Kelly RJ, Ajani JA, Kuzdzal J, Zander T, Van Cutsem E, Piessen G, et al; CheckMate 577 Investigators. Adjuvant Nivolumab in Resected Esophageal or Gastroesophageal Junction Cancer. N Engl J Med. 2021;384(13):1191-1203.

5. Eads J.R., Weitz M, Catalano P.J, Gibson M.K., Rajdev L, Khullar O, et al. A phase II/III study of perioperative nivolumab and ipilimumab in patients (pts) with locoregional esophageal (E) and gastroesophageal junction (GEJ) adenocarcinoma: Results of a safety run-in—A trial of the ECOG-ACRIN Cancer Research Group (EA2174). J Clin Oncol. 2021;39(15_Suppl):4064.

6. Hong MH, Kim HR, Park SY, Kim DJ, Lee CG, Cho J, et al. A phase II trial of preoperative chemoradiotherapy and pembrolizumab for locally advanced esophageal squamous cell carcinoma (ESCC). J Clin Oncol. 2019;37(15_Suppl):4027.

**Protocol of nICRT or nICT (n=13)**

1. Zhou M, Yang W, Xuan Y, Zou W, Wang Y, Zhang Z, et al. A study protocol of a randomized phase II trial of perioperative chemoimmunotherapy verses perioperative chemoimmunotherapy plus preoperative chemoradiation for locally advanced gastric (G) or gastroesophageal junction (GEJ) adenocarcinoma: the NeoRacing study. BMC Cancer. 2022;22(1):710.

2. Wei J, Lu X, Liu Q, Fu Y, Liu S, Li L, et al. Efficacy and Safety of Sintilimab in Combination with Concurrent Chemoradiotherapy for Locally Advanced Gastric or Gastroesophageal Junction (GEJ) Adenocarcinoma (SHARED): Study Protocol of a Prospective, Multi-Center, Single-Arm Phase 2 Trial. Cancer Manag Res. 2022;14:2007-2015.

3. He W, Wang C, Wu L, Wan G, Li B, Han Y, et al. Tislelizumab Plus Chemotherapy Sequential Neoadjuvant Therapy for Non-cCR Patients After Neoadjuvant Chemoradiotherapy in Locally Advanced Esophageal Squamous Cell Carcinoma (ETNT): An Exploratory Study. Front Immunol. 2022;13:853922.

4. Li X, Xu C, Qiu H, Chen D, Zhu K, Zhang B, et al. A single-arm, multicenter, phase II clinical study of tislelizumab plus albumin-bound paclitaxel/cisplatin as neoadjuvant therapy for borderline resectable esophageal squamous cell carcinoma. Ann Transl Med. 2022;10(5):263.

5. Shang X, Zhang W, Zhao G, Liang F, Zhang C, Yue J, et al. Pembrolizumab Combined With Neoadjuvant Chemotherapy Versus Neoadjuvant Chemoradiotherapy Followed by Surgery for Locally Advanced Oesophageal Squamous Cell Carcinoma: Protocol for a Multicentre, Prospective, Randomized-Controlled, Phase III Clinical Study (Keystone-002). Front Oncol. 2022;12:831345.

6. Yang Y, Zhu L, Cheng Y, Liu Z, Cai X, Shao J. Three-arm phase II trial comparing camrelizumab plus chemotherapy versus camrelizumab plus chemoradiation versus chemoradiation as preoperative treatment for locally advanced esophageal squamous cell carcinoma (NICE-2 Study). BMC Cancer. 2022;22(1):506.

7. Shang X, Zhao G, Liang F, Zhang C, Zhang W, Liu L, et al. Safety and effectiveness of pembrolizumab combined with paclitaxel and cisplatin as neoadjuvant therapy followed by surgery for locally advanced resectable (stage III) esophageal squamous cell carcinoma: a study protocol for a prospective, single-arm, single-center, open-label, phase-II trial (Keystone-001). Ann Transl Med. 2022;10(4):229.

8. Wang KX, Cui TY, Yang XD, Wang GQ, Jiang QS, Sun H, et al. Study on Efficacy and Safety of Low-Dose Apatinib Combined with Camrelizumab and SOX Regimen as First-Line Treatment of Locally Advanced and Unresectable Gastric/Gastroesophageal Junction Cancer: A Protocol for an Open-Label, Dose Escalation and Extension Phase Ib Clinical Trial. Onco Targets Ther. 2021;14:4859-4865.

9. Zheng Y, Liu XB, Sun HB, Xu J, Shen S, Ba YF, et al; written on Henan Cancer Hospital Thoracic Oncology Group (HCHTOG). A phase III study on neoadjuvant chemotherapy versus neoadjuvant toripalimab plus chemotherapy for locally advanced esophageal squamous cell carcinoma: Henan Cancer Hospital Thoracic Oncology Group 1909 (HCHTOG1909). Ann Transl Med. 2021;9(1):73.

10. Zheng Y, Wang Z, Yan C, Yan M, Hou Z, Zheng R, et al. Protocol for a randomized controlled trial of perioperative S-1 plus oxaliplatin combined with apatinib and camrelizumab in patients with resectable, locally advanced gastric or gastroesophageal junction adenocarcinoma. Ann Transl Med. 2020;8(24):1684.

11. Xing W, Zhao L, Fu X, Liang G, Zhang Y, Yuan D, et al; written on Henan Cancer Hospital Thoracic Oncology Group (HCHTOG). A phase II, single-centre trial of neoadjuvant toripalimab plus chemotherapy in locally advanced esophageal squamous cell carcinoma. J Thorac Dis. 2020;12(11):6861-6867.

12. Yamamoto S, Kato K, Daiko H, Kojima T, Hara H, Abe T, et al. Feasibility study of nivolumab as neoadjuvant chemotherapy for locally esophageal carcinoma: FRONTiER (JCOG1804E). Future Oncol. 2020;16(19):1351-1357.

13. Yu R, Wang W, Li T, Li J, Zhao K, Wang W, et al. RATIONALE 311: tislelizumab plus concurrent chemoradiotherapy for localized esophageal squamous cell carcinoma. Future Oncol. 2021;17(31):4081-4089.

**Gastric (G) or gastroesophageal junction (GEJ) adenocarcinoma without separate data of GEJ (n=5)**

1. Bang YJ, Van Cutsem E, Fuchs CS, Ohtsu A, Tabernero J, Ilson DH, et al. KEYNOTE-585: Phase III study of perioperative chemotherapy with or without pembrolizumab for gastric cancer. Future Oncol. 2019;15(9):943-952.

2. Homann N, Lorenzen S, Schenk M, Thuss-Patience PC, Goekkurt E, Hofheinz R.D., et al. Interim safety analysis of the DANTE trial: Perioperative atezolizumab in combination with FLOT versus FLOT alone in patients with resectable esophagogastric adenocarcinoma—A randomized, open-label phase II trial of the German Gastric Group at the AIO and SAKK. J Clin Oncol. 2020;38(15_Suppl):4549.

3. Liu Y, Han G, Li H, Zhao Y, Zhuang J, Wang G, et al. Camrelizumab combined with FOLFOX as neoadjuvant therapy for resectable locally advanced gastric and gastroesophageal junction adenocarcinoma. J Clin Oncol. 2020;38(15_Suppl):4536.

4. Wei j, Lu X, Liu Q, Fu Y, Liu S, Yang J, et al. SHARED: Efficacy and safety of sintilimab in combination with concurrent chemoradiotherapy (cCRT) in patients with locally advanced gastric (G) or gastroesophageal junction (GEJ) adenocarcinoma. J Clin Oncol. 2021;39(15_Suppl):4040.

5. Al-Batran S-E, Lorenzen S, Thuss-Patience P.C, Homann N, Schenk M, Lindig U, et al.

Surgical and pathological outcome, and pathological regression, in patients receiving perioperative atezolizumab in combination with FLOT chemotherapy versus FLOT alone for resectable esophagogastric adenocarcinoma: Interim results from DANTE, a randomized, multicenter, phase IIb trial of the FLOT-AIO German Gastric Cancer Group and Swiss SAKK. J Clin Oncol. 2022;40(16_Suppl):4003.

**ICIs in combination with chemotherapy or chemoradiotherapy without surgery (n=2)**

1. Zhang W, Yan C, Gao X, Li X, Cao F, Zhao G, et al. Safety and Feasibility of Radiotherapy Plus Camrelizumab for Locally Advanced Esophageal Squamous Cell Carcinoma. Oncologist. 2021;26(7):e1110-e1124.

2. Zhang W, Yan C, Zhang T, Chen X, Dong J, Zhao J, et al. Addition of camrelizumab to docetaxel, cisplatin, and radiation therapy in patients with locally advanced esophageal squamous cell carcinoma: a phase 1b study. Oncoimmunology. 2021;10(1):1971418.

**Retrospective studies (n=15)**

1. Lv H, Tian Y, Li J, Huang C, Sun B, Gai C, et al. Neoadjuvant Sintilimab Plus Chemotherapy in Resectable Locally Advanced Esophageal Squamous Cell Carcinoma. Front Oncol. 2022;12:864533.

2. Hong ZN, Weng K, Peng K, Chen Z, Lin J, Kang M. Neoadjuvant Immunotherapy Combined Chemotherapy Followed by Surgery Versus Surgery Alone for Locally Advanced Esophageal Squamous Cell Carcinoma: A Propensity Score-Matched Study. Front Oncol. 2021;11:797426.

3. Fan M, Dai L, Yan W, Yang Y, Lin Y, Chen K. Efficacy of programmed cell death protein 1 inhibitor in resection transformation treatment of esophageal cancer. Thorac Cancer. 2021;12(15):2182-2188.

4. Sihag S, Ku GY, Tan KS, Nussenzweig S, Wu A, Janjigian YY, et al. Safety and feasibility of esophagectomy following combined immunotherapy and chemoradiotherapy for esophageal cancer. J Thorac Cardiovasc Surg. 2021;161(3):836-843.e1.

5. Park SY, Hong MH, Kim HR, Lee CG, Cho JH, Cho BC, et al. The feasibility and safety of radical esophagectomy in patients receiving neoadjuvant chemoradiotherapy with pembrolizumab for esophageal squamous cell carcinoma. J Thorac Dis. 2020;12(11):6426-6434.

6. Ogoshi K, Satou H, Isono K, Mitomi T, Endoh M, Sugita M. Possible predictive markers of immunotherapy in esophageal cancer: retrospective analysis of a randomized study. The Cooperative Study Group for Esophageal Cancer in Japan. Cancer Invest. 1995;13(4):363-9.

7. Yang G, Su X, Yang H, Luo G, Gao C, Zheng Y, et al. Neoadjuvant programmed death-1 blockade plus chemotherapy in locally advanced esophageal squamous cell carcinoma. Ann Transl Med. 2021;9(15):1254.

8. Wu Z, Zheng Q, Chen H, Xiang J, Hu H, Li H, et al. Efficacy and safety of neoadjuvant chemotherapy and immunotherapy in locally resectable advanced esophageal squamous cell carcinoma. J Thorac Dis. 2021;13(6):3518-3528.

9. Huang B, Shi H, Gong X, Yu J, Xiao C, Zhou B, et al. Comparison of efficacy and safety between pembrolizumab combined with chemotherapy and simple chemotherapy in neoadjuvant therapy for esophageal squamous cell carcinoma. J Gastrointest Oncol. 2021;12(5):2013-2021.

10. Hong ZN, Gao L, Weng K, Huang Z, Han W, Kang M. Safety and Feasibility of Esophagectomy Following Combined Immunotherapy and Chemotherapy for Locally Advanced Esophageal Squamous Cell Carcinoma: A Propensity Score Matching Analysis. Front Immunol. 2022;13:836338.

11. Cheng J, Guo M, Yang Y, Liu Y, Hu W, Shang Q, et al. Perioperative Outcomes of Minimally Invasive Esophagectomy After Neoadjuvant Immunotherapy for Patients With Locally Advanced Esophageal Squamous Cell Carcinoma. Front Immunol. 2022;13:848881.

12. Ma X, Zhao W, Li B, Yu Y, Ma Y, Thomas M, et al. Neoadjuvant Immune Checkpoint Inhibitors Plus Chemotherapy in Locally Advanced Esophageal Squamous Cell Carcinoma: Perioperative and Survival Outcomes. Front Oncol. 2022;12:810898.

13. Yang Y, Tan L, Hu J, Li Y, Mao Y, Tian Z, et al; Esophageal Cancer Committee of Chinese Anti-Cancer Association. Safety and efficacy of neoadjuvant treatment with immune checkpoint inhibitors in esophageal cancer: real-world multicenter retrospective study in China. Dis Esophagus. 2022:doac031.

14. Xiao X, Yang YS, Zeng XX, Shang QX, Luan SY, Zhou JF, et al. The comparisons of neoadjuvant chemoimmunotherapy versus chemoradiotherapy for oesophageal squamous cancer. Eur J Cardiothorac Surg. 2022;62(1):ezac341.

15. Gu YM, Shang QX, Zhang HL, Yang YS, Wang WP, Yuan Y, et al. Safety and Feasibility of Esophagectomy Following Neoadjuvant Immunotherapy Combined with Chemotherapy for Esophageal Squamous Cell Carcinoma. Front Surg. 2022;9:851745.
